# Experimental and numerical investigation of the TBM disc cutter wear using a new tunnel boring machine laboratory simulator

**DOI:** 10.1016/j.heliyon.2024.e37148

**Published:** 2024-08-29

**Authors:** Hamid Chakeri, Mohammad Darbor, Hadi Shakeri, Hamid Mousapour, Vahid Mohajeri

**Affiliations:** Department of Mining Engineering, Sahand University of Technology, Tabriz, Iran

**Keywords:** Mechanized excavation, Wear, Excavation blade, Numerical modelling, PFC3D

## Abstract

One of the essential and practical issues on TBM performance is the wear during the excavation process of abrasive and resistant rock. The wear of the disc cutter, which is caused by the gradual and uniform reduction of the diameter of the disc cutter, is caused by the rock-machine interaction during excavation, and various factors can intensify this phenomenon and reduce the wear life of the disc cutter. To obtain a proper view of the relationship between the operating parameters of the excavation machine and the disc cutter wear, a new laboratory device has been designed and built in the mechanized excavation laboratory of the Sahand University of Technology. By using this device, the amount of wear of cutting tools in excavation machines can be obtained against rough rock samples, and from the results, the amount of wear of cutting tools of excavation machines can be minimized so that the efficiency of the excavation machine can be increased. In the current research, the laboratory wear results obtained from this device have been compared with those obtained from the numerical modelling of the same device in PFC3D discrete element software, both for the cutting blades and the sample itself. This research showed that in the first abrasion stage of the sample, the difference in the abrasion weight of the experimental study and numerical modelling for the samples varies from 0.91 to 0.768 g, and the average is 0.202 g. Also, the difference between the abrasion percentage of laboratory study and numerical simulation for the samples varies from 6 to 14 %, and the average is 10 %. In step 2 of abrasion, the difference in the abrasion weight of the experimental study and numerical modelling for the samples varies from 0.118 to 0.556 g, and the average is 0.278 g. Also, the difference in the abrasion percentage of laboratory study and numerical simulation for the samples varies from 7 to 14 %, and the average is 11 %. The results of the new tunnel boring machine laboratory simulator revealed insights into wear behavior during different stages of excavation.

## 1 Introduction

Rock cutting involves various types of interactions between the rock and the tool, resulting in damage to the tool. Zum Gahr (1987) identified plastic deformation, corrosion, cracks, and abrasion as the mechanisms responsible for this damage. This study focuses explicitly on wear, defined as the gradual loss of material from a solid body due to mechanical action [1]. In rock-cutting tools, wear typically occurs on disc cutters [2]. During rock excavation, tunnelling or mining, rock abrasivity can reduce the performance of mechanical tools due to wear. Rock abrasivity refers to the abrasion of rock materials caused by cutting tools [3]. The shield cutter load changes significantly when tunnel boring machines (TBMs) excavate soft and hard uneven strata, and the possibility of cutter damage is increased if the shield operation parameters are not optimized correctly [4]. In the absence of promptly detecting and replacing excessively worn cutters, they can not only accelerate the failure of their adjacent cutters but also increase the risk of cutter head damage [5]. It is, therefore, crucial to monitor the wear status of cutters to ensure safe and sustainable tunnel construction and cost management.

Tunnel boring machines (TBMs) are increasingly used in infrastructure construction activities as mechanical industry and automation technology develop, including hydropower projects [[6], [7], [8], [9]], railways [[10], [11], [12], [13], [14], [15]] and mining projects [16]. TBM projects require research on rock fragmentation mechanisms. The effect of disc cutter force on machine penetration was investigated using numerical simulations based on discrete element method (DEM) [[17], [18], [19], [20]] and finite element method (FEM) [21,22]. Also, some researchers studied 3D numerical modelling of polycrystalline diamond compact (PDC) to improve rock-breaking efficiency [[23], [24], [25], [26]].

Several studies examined disc cutter wear patterns during excavation, shield operation parameters, geological conditions, and cutter design. These studies include theoretical, experimental, and numerical analyses. Various theoretical and laboratory studies have been conducted on the parameters affecting the wear of the TBM disc cutter, cutter performance, and penetration rate of the excavation machine. Hassanpour et al. (2015) introduced a new empirical model for estimating penetration rate and disc cutter life in hard rock tunnel boring machines (TBMs). Based on recent tunneling projects, this empirical model predicts TBM performance and cutter wear. They also studied different ground types that were classified into seven abrasivity classes [27]. Jeong et al. (2018) investigated the effect of cutting conditions on rock chip production and size distribution during linear cutting tests in Linyi sandstone from China. Image processing provided useful information, and the size distribution parameters correlated with cutter forces and specific energy. Their study showed that penetration depth was significant in rock chip size during the excavation [28]. Cheng et al. (2018) propose a new analytical model for rock cutting force and failure surface in Polycrystalline Diamond Compact (PDC) bits used in oil drilling engineering. The model considers stress state calculations for micro-units within the rock and predicts cutting force accurately. This study showed that the increase in cut depth and cutter diameter led to a larger width of the cut, which decreased the normal force and shear force on unit width [23]. Rodríguez et al. (2021) examined a section of an actual tunnel carved through tough and abrasive rock. They investigated the rates of progress, frequency of cutter changes, and number of cutters swapped out and then analyzed how the decrease in advancement speed was impacted by both worn cutters' reduced performance and the time spent replacing them. This study showed that an increase in performance led to a higher cutter consumption, but conversely, a higher frequency of cutter replacement will lead to a higher performance [29]. Mousapour et al. (2023) examined cutting tool wear using a TBM laboratory simulation. Their studies showed that reducing the rotation speed of the cutter head from 35 to 10 rpm reduces average cutting tool wear by up to 63 %. Furthermore, reducing excavation time from 80 to 10 min reduces cutting tool wear by up to 58 %. Cutting tool wear increases with moisture content from 0 to 10 % and then decreases with an increase in moisture from 10 to 25 % [30]. Amoun and Chakeri (2023) designed and built a tunnel-boring machine simulator (TBM) to investigate the impact of various parameters on cutting tool wear. Their study showed that tool wear in coarse-grained soils is less than in fine-grained soils. The results also indicated that particle size distribution depends on silt and clay content in soil samples. When excavated materials are suitable, tool wear and torque can be reduced by 58 % and 34 %, respectively [31]. Yiqiang et al. (2023) investigated the relationship between cutting depth and disc cutter penetration of TBM in hard rock tunnels through experimental and numerical simulations. They showed that when groove cutting depth is below 4 mm, groove cutting provides minimal assistance in the disc cutter's rock-breaking process. Conversely, when the groove was deeper than 4 mm significantly aided the disc cutter's rock-breaking process [32]. Fei et al. (2024) researched the use of high-pressure water jets in combination with disc cutters for tunnel excavation in hard rock. They studied the impact of variables such as water pressure, nozzle diameter, and nozzle speed on cutting efficiency through laboratory experiments. Their findings demonstrate that high-pressure water jets are a highly effective method for breaking rock, making them ideal for industrial use due to their ability to keep pace with TBM advancement and achieve efficient rock breaking. High-pressure water jets, however, increase the ambient temperature and the water jet temperature in the tunnel, reducing the cooling effect of the cutter head and decreasing the efficiency of the TBM's construction [33]. Sabri et al. (2024) examined how wear on disc cutters impacts TBM performance metrics like thrust force, net penetration rate, and specific energy. They visually analyzed six types of worn disc cutters and devised a formula to determine the contact surface area between the cutter and rock based on cutter tip width. The findings revealed that replacing worn disc cutters enhances penetration rate while reducing thrust force, resulting in an overall increase in specific penetration by approximately 35 % [34].

Also, various numerical studies have been conducted on simulating rock cutting, crushed rock interactions, predicting cutter wear, evaluating the rock-breaking, rock-breaking mechanism and cutting performance. Labra et al. (2017) presented a hybrid discrete/finite element model for simulating rock cutting. Discrete elements represent the fractured part, while finite elements model the undamaged portion. The model accurately captures physical phenomena during cutting and efficiently computes results. It was applied to simulate rock-cutting tests using a tunnel boring machine (TBM) disc cutter [22]. Hu et al. (2020) employed LS-DYNA finite element software to model crushed rock interactions with the tipped hob. The study explored feed force, lateral force, and positive force variations at different penetration depths. Key findings include the exponential growth of forces with increasing penetration depth and the effectiveness of spherical tooth designs in reducing cutter wear. These results offer insights for selecting an optimal tipped hob to enhance rock-breaking efficiency [35]. Sabri et al. (2023) studied the impact of disc cutter wear on rock-cutting forces in Tunnel Boring Machines (TBM) and employed finite-element modelling to analyze the effects. By calibrating their models with data from linear cutting machine tests (LCM), they found that as disc cutters wear, there is an asymmetry in side forces acting on them, and these forces originate from within the cutter. Additionally, the wear of disc cutters leads to a notable rise in specific excavation energy, resulting in a substantial decrease in excavation efficiency [21]. Zhang and Zhao (2023) suggested a method for predicting cutter wear during shield tunnelling in real-time using a deep-learning model. Their proposed method can reduce the cost of cutter replacement by reducing the time needed for machine interventions [36]. Zou et al. (2023) used discrete element analyses to evaluate the rock-breaking. Under the condition of no confining pressure, crack propagation will deviate from the central axis of the cutters, which increases damage and fragmentation of the rock-breaking [37]. Ma et al. (2023) examined the rock-breaking mechanism and cutting performance of five commonly used carbide buttons, including spherical, saddle, wedge, conical, and parabolic buttons, using the three-dimensional discrete element method (DEM). Comparing simulation results with laboratory tests revealed insights into the rock indentation process. The study systematically analyzed the penetration index, specific energy, and crack propagation characteristics. Findings showed that the conical button was highly efficient at breaking rock with shallow penetration depths, while the saddle button excelled in high rock-breaking efficiency situations [20]. Mo et al. (2024) used stratal slicing to combine segmented and discrete uniaxial compressive strength (UCS) test data with geological profiles to create a sequential dataset. They show that the UCS data obtained using the proposed stratal slicing method can improve prediction accuracy compared to traditional methods and models [38].

In this study, to achieve a suitable relationship between the operating parameters of the excavation machine and the disc cutter wear, a new laboratory device was designed and built in the mechanized excavation laboratory of Sahand University of Technology. This device allows for determining the wear rate on cutting tools against rock samples, which can then be used to minimize wear and improve the performance of excavation machines. For this purpose, mineralogy and microscopic studies were done on eight different rock samples. The excavation device, specifically designed for conducting laboratory tests on rocks, most previously built devices were used for soil testing. Another advantage of this device is its horizontal excavation capability, which is a highly valuable feature. Another feature of the device is its continuous excavation capability. Additionally, the experimental wear results obtained from this device were compared with the wear results obtained from numerical modelling, both for cutting blades and rock samples.

## 2 Materials and methods

To study the wear of the disc cutter of the excavation machine, eight rock samples with different genesis were selected and using a new laboratory simulator of the tunnel boring machine, the wear rate of blades of disc cutter and the rock samples in two different penetration depths of 1 mm (the first stage of wear) and penetration depth of 2 mm (the second stage of wear) was determined.

### 2.1 Microscopic studies of rock samples

Evaluation and measurement of many minerals and rocks in manual samples is impossible due to their fine nature. Thin section studies and polarizing microscopes must be used to examine such samples. For example, in a sample of rock like granite, where its minerals are visible as dark and light grains to the naked eye, constituent minerals can be somewhat identified through macroscopic examinations, allowing for identifying and naming the rock sample. However, this cannot be done for all types of rocks because the constituent minerals are often fine-grained and not distinguishable to the naked eye. For instance, basalt or fine-crystalline limestone can be mentioned. To thoroughly examine such rocks and, in general, all rocks with a microscope, thin sections must be prepared so that light can easily pass through transparent mineral grains in thin sections. In thin sections, not only can the types of minerals in each rock be identified, but also the arrangement and shape of the grains, the texture of the rock, the cement connecting the grains, and even the relative crystallization time of each mineral can be determined.

**Fig. 1:**
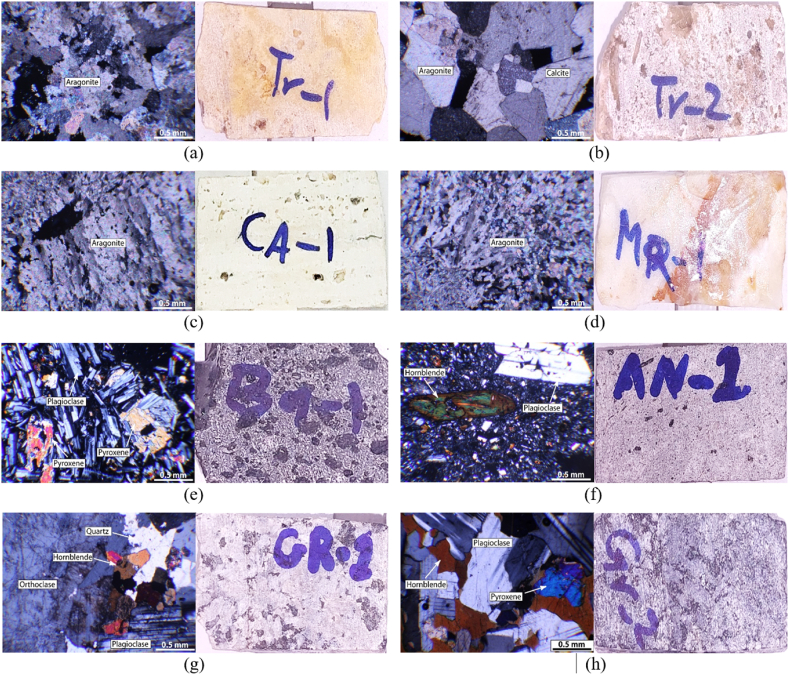
Microscopic characteristics of rock samples; a) TR-1 b) TR-2 c) CA-1 d) MR-1 e) BA-1 f) AN-1 g) GR-1 h) GR-2.

In this study, thin section studies were conducted on eight different rock samples, including 3 different travertine samples, one marble sample, two quartz syenite samples, one andesite sample and one gabbro sample. The microscopic characteristics of the studied samples are described in Table 1 and Fig. 1. Also, the mechanical properties of the rock samples are illustrated in Table 2.

**Table 1:** Macroscopic properties of studied rock samples.

Sample	Rock Type	Main minerals	Secondary minerals	Percentage of minerals	Texture	Porosity	Crystal size
**Travertine (TR-1)**	Travertine	Aragonite	–	Aragonite (95 %)	Crystalline and porous texture	About 5 %	Coarse to fine crystal
**Travertine (TR-2)**	Travertine	Aragonite	Calcite	Aragonite (60 %) and Calcit (20 %)	Crystalline and porous texture	About 20 %	Coarse to fine crystal
**Travertine (CA-1)**	Travertine	Aragonite	–	Aragonite (90 %)	Crystalline and porous texture	About 10 %	Coarse to fine crystal
**Onyx marble (MR-1)**	Marble	Aragonite	Anhydrite	Aragonite (90 %) and Anhydrite (15 %)	Crystalline	About 5 %	The size of the crystals is variable
**Basalt (BA-1)**	Quartz syenite	Feldspar (plagioclas), pyroxene (clino and orthopyroxene), olivine	Opec minrals	Feldspar (50 %), pyroxene (25 %), olivine (10 %)	Porphyry, aphantic and microlithic porphyric textures	High porosity	Fine crystal
**Andesite (AN-1)**	Andesite	Plagioclase, Hornblende	Calcite, opec minerals, volcanic glass	Plagioclase (50 %), hornblende(30 %)	Porphyry and cavity texture	Low porosity (2 %)	Coarse to fine crystal
**Quartz syenite (GR-1)**	Quartz syenite	Alkali feldspar (orthoclase), plagioclase	Quartz, biotite, amphibole, calcite, clay minerals, opac minerals, muscovite, epidote, apatite and zircon	Alkali feldspar (orthoclase) (50 %) and plagioclase (30 %)	Granular hydromorphic texture	No	Coarse crystal
**Hornblende gabbro (GR-2)**	Gabbro	plagioclase, pyroxene, hornblende and olivine	Opec minerals, cercite, talc, chlorite and calcite	Plagioclase (50 %), pyroxene (20 %), hornblende (15 %) and olivine(10 %)	Granular hydromorphic texture	No	Coarse crystal

**Table 2:** The mechanical properties of the rock samples.

Sample	Modulus of elasticity (GPa)	Poisson ratio	Tensile strength (MPa)	Compressive strength (MPa)	Schmidt hardness	Density ($\frac{kg}{m^{3}}$)
**Andesite (AN-1)**	13.7	0.24	5.6	42	43.65	2620
**Travertine (CA-1)**	25	0.23	11.9	133	45.43	2580
**Travertine (TR-1)**	12	0.26	4.3	43	32.93	2600
**Travertine (TR-2)**	31	0.25	7.8	47	48.52	2550
**Onyx marble (MR-1)**	12.4	0.27	7.6	48	40.85	2560
**Quartz syenite (GR-1)**	19	0.22	6.2	76	43.11	2750
**Hornblende gabbro (GR-2)**	17	0.27	7.1	59	37.62	2710
**Basalt (BA-1)**	18	0.27	7.1	59	38.87	2730

## 3 Introducing the new laboratory simulation of the tunnel boring machine

**Fig. 2:**
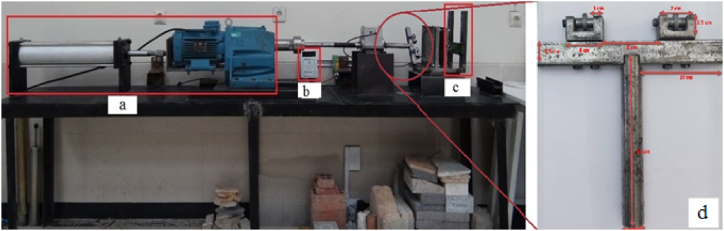
The laboratory simulation of tunnel boring machine a) pneumatic jack, b) torque meter, c) load cell and, d) Dimensions of different parts of blade wear-measuring device.

**Fig. 3:**
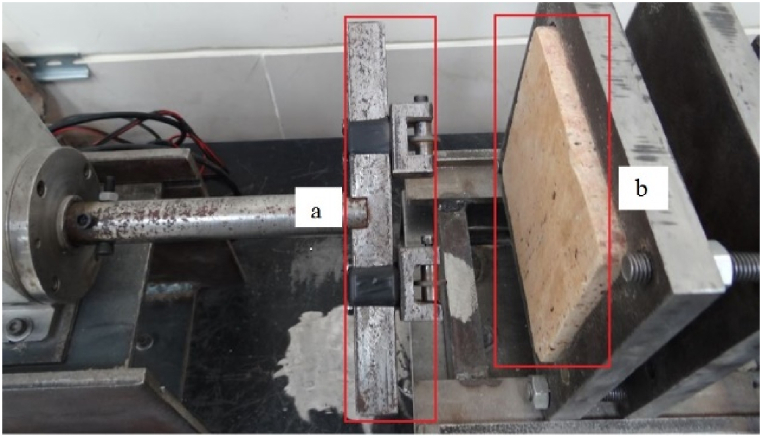
a) T-shaped tool representing the cutter head of the mechanized excavation machine b) location of the rock sample.

In this study, a new laboratory device was designed and built in the mechanized excavation laboratory of Sahand University of Technology. This device allows for determining the wear rate on cutting tools against rock samples, which can then be used to minimize wear and improve the performance of excavation machines. Fig. 2 shows the position of the torque meter (Fig. 2-a) and the load cell for measuring the forces applied to the sample (Fig. 2-b) in the new tunnel boring machine laboratory simulator. In Fig. 2-d, the blade wear measurement device is composed of a rotating axis with two cutting disks placed at variable distances. The dimensions of different parts of this device are shown. The disks (blades) diameter is 2 cm, and their thickness is 0.4 cm. The axis on which the blades are placed also has an inner diameter of 0.5 cm and an outer diameter of 0.5 cm. Also, Fig. 3 shows the location of the rock sample (worn) and the T-shaped tool, represents the head of the mechanized excavation machine.

The device shown in Fig. 2-d rotates at a constant speed at two different penetration depths of 1 mm (for measuring the wear of step 1) and 2 mm (for measuring the wear of step 2) on various rock samples. The blades' weights before and after wear are measured, and the results for the wear of step 1 are presented in Table 3, while the results for the wear of step 2 are presented in Table 4. The weight loss of the sample is also equal to the difference in weight of the rock sample before and after wear.

**Table 3:** Step 1 of wear of blades for different rock samples in the test.

Sample	Blade weight 1		Blade weight 2		The weight of the crushed sample (g)
	Initial weight (g)	After test (g)	Initial weight (g)	After test (g)	
**Andesite (AN-1)**	14.300	14.281	14.812	14.789	1.240
**Travertine (CA-1)**	14.791	14.782	14.898	14.888	1.955
**Travertine (TR-1)**	15.170	15.149	14.539	14.527	1.101
**Travertine (TR-2)**	14.953	14.945	14.895	14.889	5.645
**Onyx marble (MR-1)**	14.814	14.800	15.162	15.157	1.230
**Quartz syenite (GR-1)**	14.569	14.543	15.136	15.114	1.070
**Hornblende gabbro (GR-2)**	14.697	14.660	14.825	14.808	1.662
**Basalt (BA-1)**	14.627	14.610	14.199	14.170	1.586

**Table 4:** Steps 2 of wear of blades for different samples of rock in the test.

Sample	Blade weight 1		Blade weight 2		The weight of the crushed sample (g)
	Initial weight (g)	After test (g)	Initial weight (g)	After test (g)	
**Andesite (AN-1)**	14.3	14.260	14.812	14.770	3.619
**Travertine (CA-1)**	14.791	14.775	14.898	14.880	4.100
**Travertine (TR-1)**	15.170	15.145	14.539	14.520	2.792
**Travertine (TR-2)**	14.953	14.940	14.895	14.884	5.562
**Onyx marble (MR-1)**	14.814	14.795	15.162	15.145	1.296
**Quartz syenite (GR-1)**	14.569	14.538	15.136	15.112	1.195
**Hornblende gabbro (GR-2)**	14.697	14.602	14.825	14.800	1.686
**Basalt (BA-1)**	14.627	14.564	14.199	14.159	1.782

## 4 Numerical modelling of disc cutter and sample wear

**Fig. 4:**
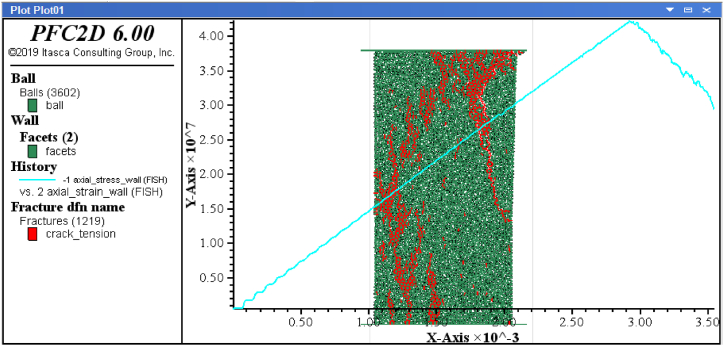
Specimen under uniaxial compressive loading for Andesite 1.

**Fig. 5:**
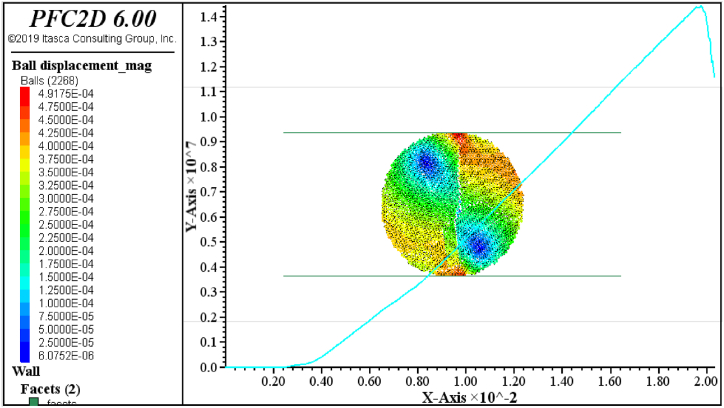
Specimen under Brazilian testing for Andesite 1.

The PFC3D software is based on the discrete element method (DEM). Therefore, all assumptions in this program correspond to the same assumptions of the discrete element method. Unlike most existing programs where soil and rock parameters, such as cohesion, friction angle, elastic modulus, etc., can be directly input, in PFC3D, these parameters cannot be directly specified. Instead, calibration is necessary by modelling uniaxial tests (conducted in the laboratory on local rock) using PFC3D and obtaining the stress-strain curve from experimental results. The calibration data is provided in Table 5. Calibration was performed using uniaxial compressive strength tests and Brazilian tests. Fig. 4 shows a specimen under uniaxial compressive loading for Andesite 1, along with the axial stress-strain curve. Additionally, Fig. 4 illustrates the microcracks generated in the specimen during uniaxial testing. In Fig. 5, the specimen is subjected to Brazilian testing for Andesite 1, showing the stress-strain diagram. Furthermore, Fig. 5 presents the particle displacement resulting from the uniaxial Brazilian test.

**Table 5:** Calibration data obtained from the samples.

Sample	Modulus of elasticity (GPa)	Poisson ratio	Tensile strength (MPa)	Compressive strength (MPa)
**Andesite (AN-1)**	14	0.24	5.8	42
**Travertine (CA-1)**	25	0.23	11.9	133
**Travertine (TR-1)**	7	0.26	4.3	43
**Travertine (TR-2)**	31	0.25	7.7	47
**Onyx marble (MR-1)**	12	0.27	7.7	48
**Quartz syenite (GR-1)**	19	0.22	6.2	76
**Hornblende gabbro (GR-2)**	17	0.27	7.1	59
**Basalt (BA-1)**	17	0.27	7.1	59

**Fig. 6:**
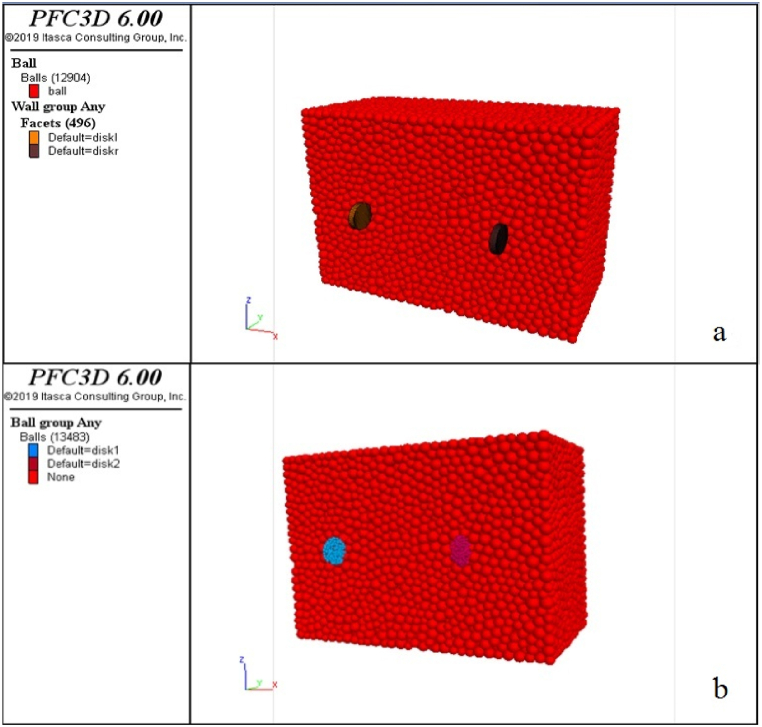
Modelling geometry including rock sample and cutting blades before the start of wear test; a). The blades are modelled as walls, b). Blades are modelled as particles.

This method uses an explicit numerical technique that examines particle interactions through contact and particle movement. Considering the calibrated samples, wear tests are numerically performed using the PFC3D software, and the amount of abrasion on the samples is determined. The geometry of the modelling, including the rock sample and cutting blades before wear initiation, is shown in Fig. 6. To determine the amount of wear on the blades, the blades are modelled in two forms: once as a wall (Fig. 6-a) and once as particles similar to the rock sample (Fig. 6-b).

**Fig. 7:**
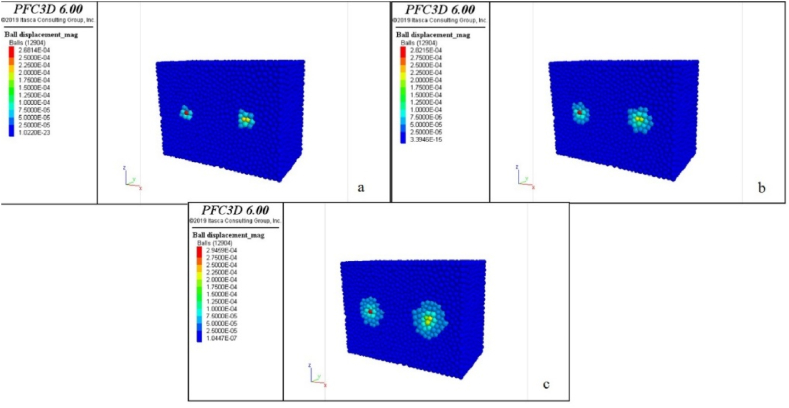
Particle displacement output after the start of abrasion and the spread of the abrasion range in the sample after a) 1 s, b) 2 s, and c) 3 s of abrasion.

With the initiation of the wear test, particles of the sample and blades start to separate from each other. The number of modelled particles decreases as each particle exits the model's domain. Considering the known number of modelled particles for the sample and each of the blade before the start of the test, it is possible to determine the number of particles after wear for each of the blade and the sample. By multiplying the difference in the number of particles for each blade and sample before and after wear by the weight of each particle (m=ρV, m: the weight of each particle (kg), ρ:density ($\frac{kg}{m^{3}}$) and V: the volume of each particle ($m^{3}$), $V=\frac{4}{3}\pi r^{3}$, r: the radius of each particle (m)), the amount of wear weight for the blades and sample can be obtained. The results in two different penetration depths of 1 mm (to measure the wear of step 1) and penetration depth of 2 mm (to measure wear of step 2) in Table 6, Table 7. Also, Fig. 7 shows the output of particle displacement after 1, 2, and 3 s from the start of abrasion and the spread of abrasion range in the sample.

**Table 6:** The weight of the wear of step 1 in penetration depth of 1 mm for different rock samples in numerical modelling.

Sample	Blade weight 1		Blade weight 2		The weight of the crushed sample (g)
	Initial weight (g)	After test (g)	Initial weight (g)	After test (g)	
**Andesite (AN-1)**	14.300	14.275	14.812	14.782	1.105
**Travertine (CA-1)**	14.791	14.775	14.898	14.881	1.812
**Travertine (TR-1)**	15.170	15.144	14.539	14.521	0.961
**Travertine (TR-2)**	14.953	14.938	14.895	14.880	4.877
**Onyx marble (MR-1)**	14.814	14.794	15.162	15.152	1.129
**Quartz syenite (GR-1)**	14.569	14.537	15.136	14.106	0.973
**Hornblende gabbro (GR-2)**	14.697	14.656	14.825	14.798	1.571
**Basalt (BA-1)**	14.627	14.604	14.199	14.164	1.442

**Table 7:** The weight of the wear of step 2 in penetration depth of 2 mm for different rock samples in numerical modelling.

Sample	Blade weight 1		Blade weight 2		The weight of the crushed sample (g)
	Initial weight (g)	After test (g)	Initial weight (g)	After test (g)	
**Andesite (AN-1)**	14.300	14.253	14.812	14.763	3.355
**Travertine (CA-1)**	14.791	14.768	14.898	14.876	3.690
**Travertine (TR-1)**	15.170	15.140	14.539	14.511	2.538
**Travertine (TR-2)**	14.953	14.934	14.895	14.875	5.006
**Onyx marble (MR-1)**	14.814	14.786	15.162	15.134	1.178
**Quartz syenite (GR-1)**	14.569	14.529	15.136	15.101	1.032
**Hornblende gabbro (GR-2)**	14.697	14.592	14.825	14.793	1.472
**Basalt (BA-1)**	14.627	14.554	14.199	14.153	1.540

## 5 Comparison of experimental tests and numerical modelling results

In this section, the results obtained from the wear test numerical modelling in PFC3D are compared with those obtained from the actual abrasion tests on samples in the laboratory. In Table 8, Table 9**,** for the wear of step 1, the difference in wear and the percentage difference in wear for each cutting blade and the sample are provided and compared with each other. According to Tables 8 and in step 1 of the wear for blade 1, the weight difference in wear between numerical modelling and experimental study for rock samples ranged from 4 to 7g, and the average was 6g. Additionally, the percentage difference in wear between numerical modelling and experimental study for rock samples ranged from 12 to 93 %, and the average was 42 %. According to Tables 8 and in step 1 of wear for blade 2, the weight difference in wear between numerical modelling and experimental study for samples ranged from 5 to 10g, and the average was 7g. Additionally, the percentage difference in wear between numerical modelling and experimental study for samples ranged from 20 to 149 %, and the average was 64 %. Also, according to Tables 9 and in step 1 of abrasion for the rock samples, the weight difference in abrasion between numerical modelling and experimental study ranged from 0.91 to 0.768g, and the average was 2.02g. Additionally, the percentage difference in abrasion between numerical modelling and experimental study for samples ranged from 6 to 14 %, and the average was 10 %.

**Table 8:** Comparison of the wear values of step 1 of numerical modelling and experimental study in blades 1 and 2.

Sample	Wear of blade 1 (experimental study) (g)	Wear of blade 2 (experimental study) (g)	Wear of blade 1 (numerical modelling) (g)	Wear of blade 2 (numerical modelling) (g)	Comparison of laboratory wear and numerical modelling	
					Percentage of wear difference for blade 1 (%)	Percentage of wear difference for blade 2 (%)
**Andesite (AN-1)**	19	23	25	30	30	32
**Travertine (CA-1)**	9	10	16	17	82	74
**Travertine (TR-1)**	21	12	26	18	22	48
**Travertine (TR-2)**	8	6	15	15	93	149
**Onyx marble (MR-1)**	14	5	20	10	42	91
**Quartz syenite (GR-1)**	26	22	32	30	22	34
**Hornblende gabbro (GR-2)**	37	17	41	27	12	61
**Basalt (BA-1)**	17	29	23	35	34	20

**Table 9:** Comparison of the wear values of step 1 of numerical modelling and experimental study in the different rock samples.

Sample	Abrasion (experimental study) (g)	Abrasion (numerical modelling) (g)	Comparison of laboratory abrasion and numerical modelling	
			The difference in abrasion weight (g)	The difference in abrasion weight (%)
**Andesite (AN-1)**	1.240	1.105	0.135	11
**Travertine (CA-1)**	1.955	1.812	0.143	7
**Travertine (TR-1)**	1.101	0.961	0.140	13
**Travertine (TR-2)**	5.645	4.877	0.768	14
**Onyx marble (MR-1)**	1.230	1.129	0.101	8
**Quartz syenite (GR-1)**	1.070	0.973	0.970	9
**Hornblende gabbro (GR-2)**	1.662	1.571	0.910	6
**Basalt (BA-1)**	1.586	1.442	0.144	9

Table 10 illustrate the amount of difference in wear of step 2 and the percentage of difference in wear of step 2 for each cutting blade and rock sample. the results are compared with each other. According to Tables 10 and in the step 2 of the wear on blade 1, the difference between the wear weight of numerical modelling and experimental study varies from 5 to 10g, and the average is 8g. Also, the difference in wear percentage between numerical modelling and experimental study varies from 11 to 47 %, and the average is 29 %. According to Tables 10 and in the step 2 of the wear on blade 2, the weight difference between wear numerical modelling and experimental study ranges from 4 to 11g, and the average is 8g. Also, the percentage difference in wear between numerical modelling and experimental study ranges from 18 to 81 %, and the average is 40 %. According to Table 11, in the step 2 of the wear of the sample, the weight difference between abrasion numerical modelling and experimental study ranges from 0.0 to 0.6g, and the average is 0.2g. Also, the percentage difference in abrasion between numerical modelling and experimental study ranges from 7 to 14 %, and the average is 11 %.

**Table 10:** Comparison of wear values of step 2 of numerical modelling and experimental study in blades 1 and 2.

Sample	Wear of blade 1 (experimental study) (g)	Wear of blade 2 (experimental study) (g)	Wear of blade 1 (numerical modelling) (g)	Wear of blade 2 (numerical modelling) (g)	Comparison of laboratory wear and numerical modelling	
					Percentage of wear difference for blade 1 (%)	Percentage of wear difference for blade 2 (%)
**Andesite (AN-1)**	40	42	47	49	18	18
**Travertine (CA-1)**	16	18	23	22	46	25
**Travertine (TR-1)**	25	19	30	28	18	46
**Travertine (TR-2)**	13	11	19	20	46	81
**Onyx marble (MR-1)**	19	17	28	28	47	62
**Quartz syenite (GR-1)**	31	24	40	35	28	44
**Hornblende gabbro (GR-2)**	95	25	105	32	11	30
**Basalt (BA-1)**	63	40	73	46	16	14

**Table 11:** Comparison of wear values of step 2 of numerical modelling and experimental study in the different rock samples.

Sample	Abrasion (experimental study) (g)	Abrasion (numerical modelling) (g)	Comparison of laboratory abrasion and numerical modelling	
			The difference in abrasion weight (g)	The difference in abrasion weight (%)
**Andesite (AN-1)**	3.619	3.355	0.264	7
**Travertine (CA-1)**	4.100	3.690	0.410	10
**Travertine (TR-1)**	2.792	2.538	0.254	9
**Travertine (TR-2)**	5.562	5.006	0.556	10
**Onyx marble (MR-1)**	1.296	1.178	0.118	9
**Quartz syenite (GR-1)**	1.195	1.032	0.163	14
**Hornblende gabbro (GR-2)**	1.686	1.472	0.214	13
**Basalt (BA-1)**	1.782	1.540	0.242	14

In Fig. 8, Fig. 9, Fig. 10, Fig. 11, Fig. 12, Fig. 13, Fig. 14, Fig. 15, Fig. 16, Fig. 17, Fig. 18, wear values of blades in the step 1 of wear and step 2 of wear in numerical modelling is compared with experimental results for different rock samples. The obtained results revealed that the new laboratory simulator of the tunnel boring machine can accurately estimate the amount of wear of cutting blades.

**Fig. 8:**
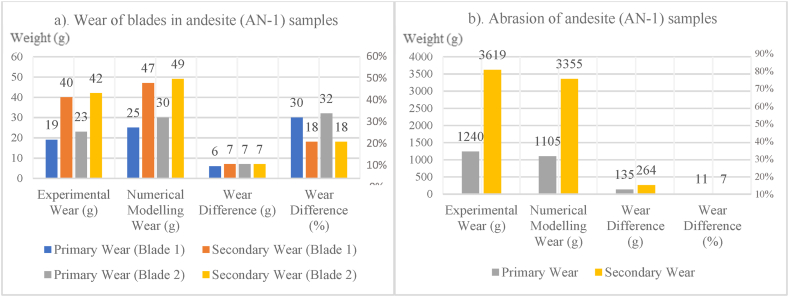
Comparing the wear values of numerical modelling and experimental study in andesite (AN-1) samples: (a) wear of blades and (b) abrasion of rock samples.

**Fig. 9:**
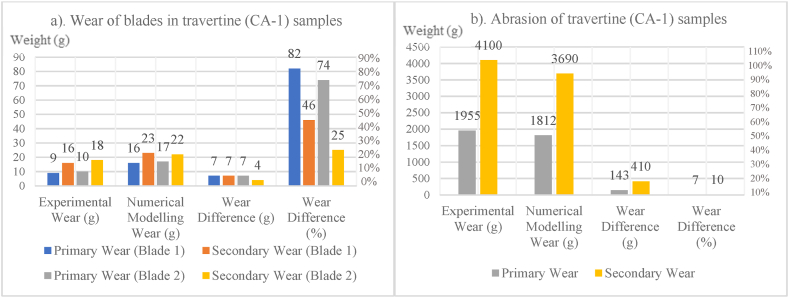
Comparing the wear values of numerical modelling and experimental study in travertine (CA-1) samples: (a) wear of blades and (b) abrasion of rock samples.

**Fig. 10:**
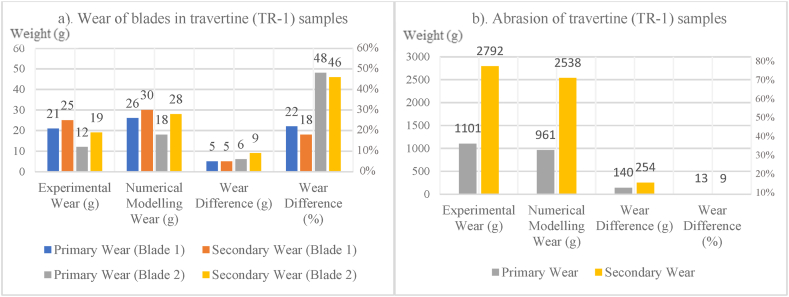
Comparing the wear values of numerical modelling and experimental study in travertine (TR-1) samples: (a) wear of blades and (b) abrasion of rock samples.

**Fig. 11:**
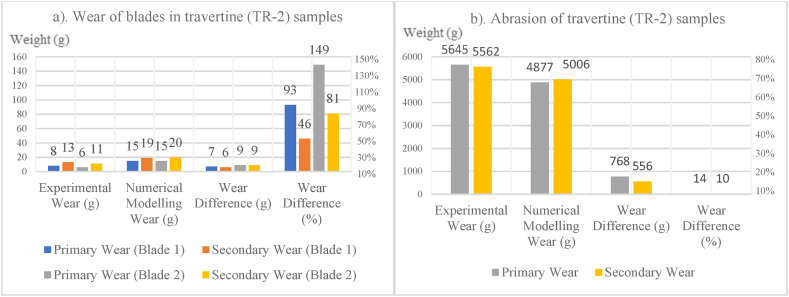
Comparing the wear values of numerical modelling and experimental study in travertine (TR-2) samples: (a) wear of blades and (b) abrasion of rock samples.

**Fig. 12:**
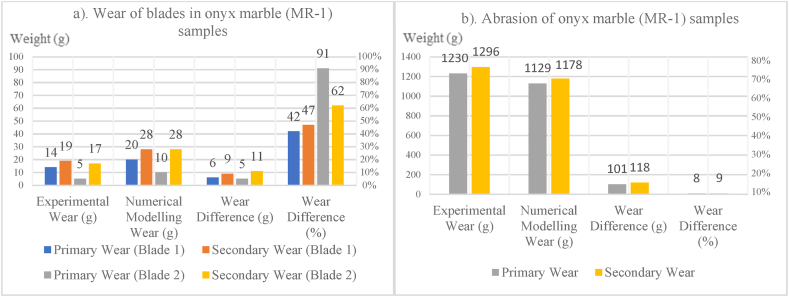
Comparing the wear values of numerical modelling and experimental study in onyx marble (MR-1) samples: (a) wear of blades and (b) abrasion of rock samples.

**Fig. 13:**
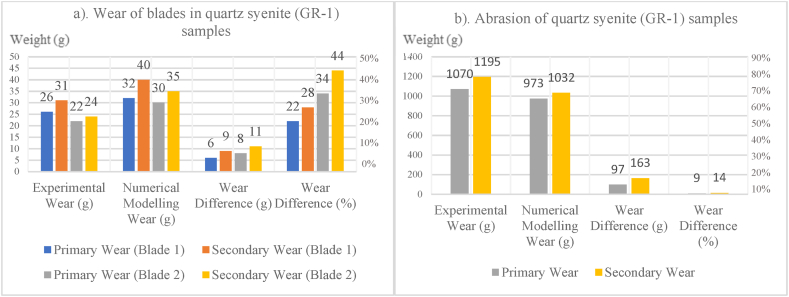
Comparing the wear values of numerical modelling and experimental study in quartz syenite (GR-1) samples: (a) wear of blades and (b) abrasion of rock samples.

**Fig. 14:**
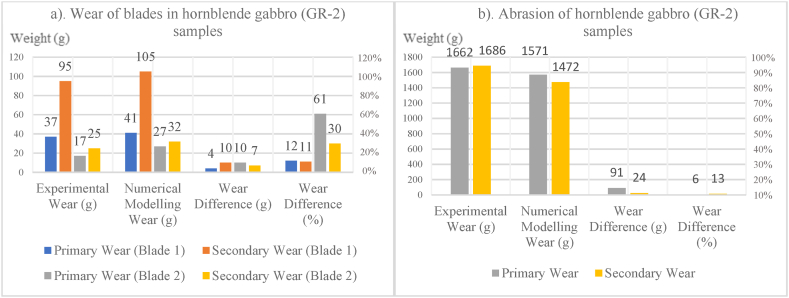
Comparing the wear values of numerical modelling and experimental study in hornblende gabbro (GR-2) samples: (a) wear of blades and (b) abrasion of rock samples.

**Fig. 15:**
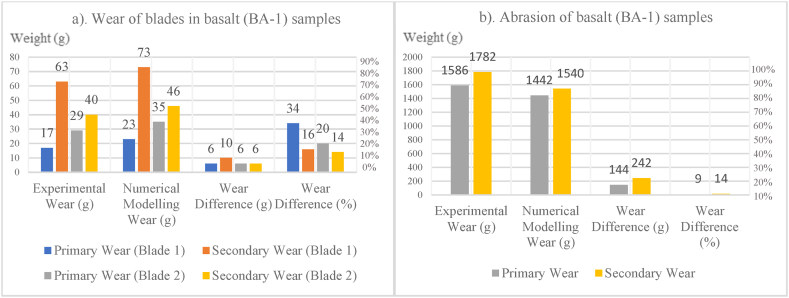
Comparing the wear values of numerical modelling and experimental study in basalt (BA-1) samples: (a) wear of blades and (b) abrasion of rock samples.

**Fig. 16:**
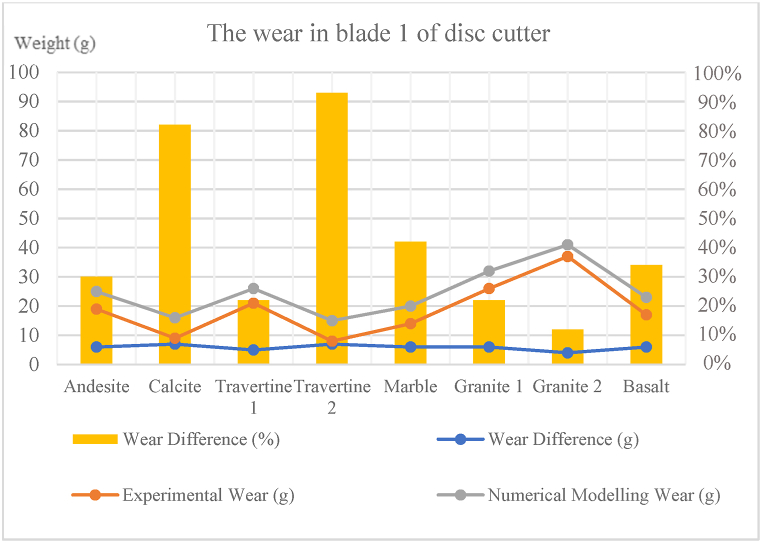
Comparing the wear of blade 1 of disc cutter in numerical modelling and experimental study for different rock samples.

**Fig. 17:**
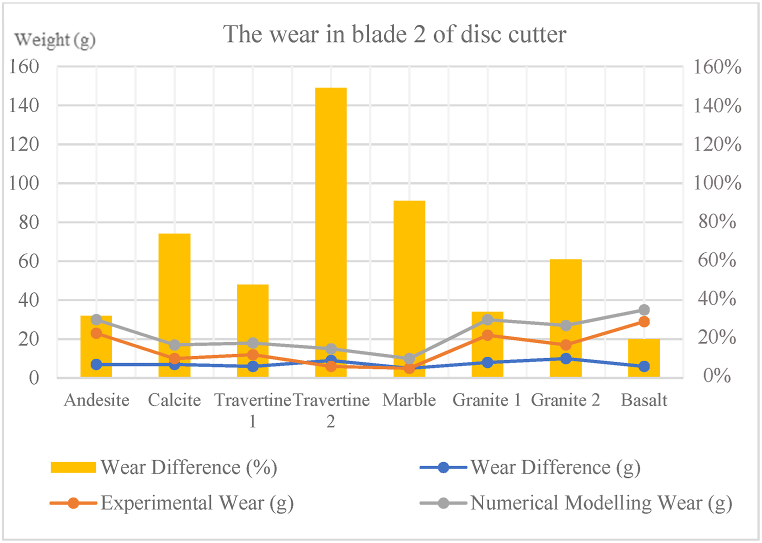
Comparing the wear of blade 2 of disc cutter in numerical modelling and experimental study for different rock samples.

**Fig. 18:**
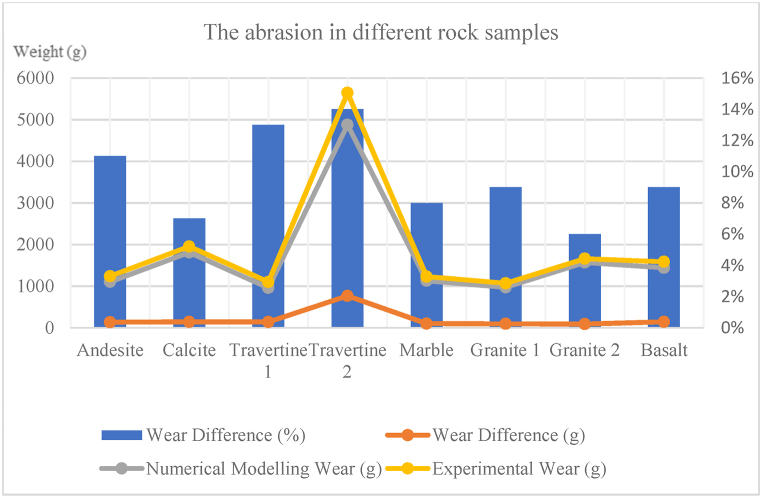
Comparing the abrasion of the different rock samples in numerical modelling and experimental study.

## 6 Results and discussion

In recent years, with the advancement of the tunnelling industry, the use of mechanized tunnel boring machines for excavating long tunnels, mountain tunnels, urban tunnels, infrastructure tunnels, and even underground mines has become common and somewhat inevitable due to the high excavation speed and suitable safety conditions. Because this method is much more expensive than conventional tunnelling methods, monitoring is necessary to ensure that unforeseen problems and unexpected events do not affect TBM performance or project planning in the future. The wear of disc cutters during the excavation process in hard and abrasive rocks is one of the significant issues affecting TBM performance. In this study, a new laboratory device was designed and built at the mechanized excavation laboratory of Sahand University of Technology. This device allows for determining the wear rate on cutting tools against rock samples, which can then be used to minimize wear and improve the performance of excavation machines. In the present study, the wear results of experimental tests obtained from this device were compared with those obtained from numerical modelling of this device in PFC3D software, both for cutting blades and rock samples. The most important results obtained from this study are presented below.

1 In most previous studies, a limited variety of rocks and different dimensions of rock samples have been used for conducting linear cutting tests. This study used 8 types of rocks with origins in sedimentary, plutonic, and volcanic sources in wear tests. Some of the chosen specimens contain minerals that exhibit high levels of abrasiveness. These rocks are often harder and more resistant to wear and erosion. On the other hand, some of the rock specimens contain minerals that have less abrasiveness. These rocks are typically softer and more susceptible to wear and erosion. Including both types of rocks in the testing process allows for a comprehensive understanding of how different mineral compositions can influence the physical properties and behavior of rocks.
2 The purpose of microscopic studies and determining the equivalent quartz content for all rock samples is to examine the presence of minerals with high Mohs hardness and determine other rock properties such as texture, crystal size, and porosity. After microscopic studies and determining the equivalent quartz content of the rock, it can be understood that the equivalent quartz content can effectively predict cutting disc wear.
3 In the step 1 of the wear on blade 1, the difference in disc cutter wear weight between numerical modelling and experimental study for different rock samples ranged from 4 to 7g and had an average of 6g. Additionally, the percentage difference in disc cutter wear between numerical modelling and experimental study for different rock samples ranged from 12 to 93 %, with an average of 42 %.
4 In the step 1 of the wear on blade 2, the difference in disc cutter wear weight between numerical modelling and experimental study of different rock samples ranged from 5 to 10g with an average of 7g. The percentage difference in disc cutter wear between numerical modelling and experimental study for different rock samples ranged from 20 to 149 %, with an average of 64 %.

5- In the step 1 of abrasion for the rock samples, the abrasion weight difference between numerical modelling and experimental study for different rock samples ranged from 0.091 to 0.768g, with an average of 0.202g. The percentage difference in abrasion between numerical modelling and experimental study for different rock samples ranged from 6 to 14 %, with an average of 10 %.

6 In the step 2 of the wear on blade 1, the difference in disc cutter wear weight between numerical modelling and experimental study for different rock samples ranged from 5 to 10g with an average of 8g. The percentage difference in disc cutter wear between numerical modelling and experimental study for different rock samples ranged from 11 to 47 %, with an average of 29 %.
7 In the step 2 of the wear on blade 2, the difference in disc cutter wear weight between numerical modelling and experimental study for different rock samples ranged from 4 to 11g with an average of 8g. The percentage difference in disc cutter wear between numerical modelling and experimental study for different rock samples ranged from 18 to 81 %, with an average of 40 %.

8- In the step 2 of abrasion for the rock samples, the abrasion weight difference between numerical modelling and experimental study for different rock samples ranged from 0.118 to 0.556g, with an average of 0.278g. The percentage difference in abrasion between numerical modelling and experimental study for different rock samples ranged from 7 to 14 %, with an average of 11 %.

9 Based on the findings of this research, it can be understood that Brazilian tensile strength shows a better correlation with cutting disc wear values among the essential mechanical properties of the examined rocks. Additionally, parameters such as uniaxial compressive strength and modulus of elasticity can provide a good indication of predicting cutting disc wear. However, determining rocks' mineralogical and physical properties, such as texture, crystal size, and porosity, alongside their mechanical properties, is crucial in predicting rock wear.
10 The findings of the experimental and numerical results of this research for TBM design and operation, as well as the potential for improving disc cutter wear resistance can be used as follows:
a). The possibility of choosing the appropriate penetration rate considering its optimal efficiency using the rock's strength parameters and consequently reducing energy consumption and increasing the life of the cutter head. b). The possibility of selecting the operational parameters of the device to maximize the penetration rate. c). Predicting the life of the cutter head and consequently the possibility of reducing the operation time and costs by timely replacement of cutter head and preventing a decrease in the efficiency of the excavation device. d). The possibility of redesigning the arrangement of cutter heads according to their performance, efficiency, and optimal life on the cutter head.

The results of this research showed that the new laboratory simulator of the tunnel boring machine can accurately estimate the amount of wear of cutting blades and can be used for future studies related to evaluating the wear of cutting tools. Also, results of the three-dimensional numerical model of the discrete element presented with PFC3D software showed that numerical modelling of this research can estimate the amount of wear of the cutting tools and the crushing of the samples, and the results obtained from it are in relatively good agreement with the experimental results.

## Funding

This research did not receive any specific grant from funding agencies in the public, commercial, or not-for-profit sectors.

## Data availability statement

Data included in article/supp. material/referenced in article.

## Ethics declarations

Review and/or approval by an ethics committee was not needed for this study because it does not include any human or animal participation.

## CRediT authorship contribution statement

**Hamid Chakeri:** Data curation, Conceptualization. **Mohammad Darbor:** Data curation, Conceptualization. **Hadi Shakeri:** Data curation, Conceptualization. **Hamid Mousapour:** Data curation, Conceptualization. **Vahid Mohajeri:** Data curation, Conceptualization.

## Declaration of competing interest

The authors declare that they have no known competing financial interests or personal relationships that could have appeared to influence the work reported in this paper.
